# Evolution and expression analysis reveal the potential role of the HD-Zip gene family in regulation of embryo abortion in grapes (*Vitis vinifera* L.)

**DOI:** 10.1186/s12864-017-4110-y

**Published:** 2017-09-21

**Authors:** Zhiqian Li, Chen Zhang, Yurui Guo, Weili Niu, Yuejin Wang, Yan Xu

**Affiliations:** 1 College of Horticulture, Northwest A&F University, Yangling, Shaanxi People’s Republic of China; 2 State Key Laboratory of Crop Stress Biology in Arid Areas, Northwest A&F University, Yangling, Shaanxi People’s Republic of China; 3 Key Laboratory of Horticultural Plant Biology and Germplasm Innovation in Northwest China, Ministry of Agriculture, Yangling, Shaanxi People’s Republic of China

**Keywords:** Homeobox, HD-Zip, *Vitis vinifera*, seedless grape, embryo abortion

## Abstract

**Background:**

The HD-Zip family has a diversity of functions during plant development. In this study, we identify 33 HD-Zip transcription factors in grape and detect their expressions in ovules and somatic embryos, as well as in various vegetative organs.

**Results:**

A genome-wide survey for *HD-Zip* transcription factors in *Vitis* was conducted based on the 12 X grape genome (*V. vinifera* L.). A total of 33 members were identified and classified into four subfamilies (I-IV) based on phylogeny analysis with *Arabidopsis*, rice and maize. *VvHDZs* in the same subfamily have similar protein motifs and intron/exon structures. An evaluation of duplication events suggests several *HD-Zip* genes arose before the divergence of the grape and *Arabidopsis* lineages. The 33 members of *HD-Zip* were differentially expressed in ovules of the stenospermic grape, Thompson Seedless and of the seeded grape, Pinot noir. Most have higher expressions during ovule abortion in Thompson Seedless. In addition, transcripts of the *HD-Zip* family were also detected in somatic embryogenesis of Thompson Seedless and in different vegetative organs of Thompson Seedless at varying levels. Additionally, VvHDZ28 is located in the nucleus and had transcriptional activity consistent with the typical features of the HD-Zip family. Our results provide a foundation for future grape *HD-Zip* gene function research.

**Conclusions:**

The identification and expression profiles of the HD-Zip transcription factors in grape, reveal their diverse roles during ovule abortion and organ development. Our results lay a foundation for functional analysis of grape HDZ genes.

**Electronic supplementary material:**

The online version of this article (10.1186/s12864-017-4110-y) contains supplementary material, which is available to authorized users.

## Background

Grapevine (*Vitis* L.) is one of the world’s most economically important, high-value, fruit crops. It is cultivated for the production of wine, table grapes, juices, distilled liquors and dry raisins. Where the fruit is eaten whole - either fresh or dried - seedlessness is one of the characteristics most appreciated by consumers. Double fertilization and embryogenesis are key reproductive process in higher plants [1]. Two kinds of seedless grapes have been characterized, stenospermocarpic and parthenocarpic. In stenospermocarpy, embryogenesis stops after double fertilization whereas in parthenocarpy double fertilization does not occur [2]. A large body of research using hormones and genes has been conducted to elucidate the mechanisms of ovule/embryo abortion in stenospermocarpic grapes [3–5]. However, the molecular basis for ovule abortion remains ambiguous.

Transcription factors are regulatory proteins which play various roles in transcriptional modulating of gene expression during plant development. They can binding to specific cis-acting elements, which existed in the promoter region of the target genes and regulate their expressions at transcription level [6]. The HD-Zip family contains a large number of transcription factors that seem be unique to the plant kingdom [7]. The HD- Zip family can be classified into four subfamilies (I - IV) in *Arabidopsis* [8], maize [9] and rice [10]. Transcription factors in HD-Zip family have Homeobox domain (HD) and a leucine zipper motif (LZ) downstream [11, 12]. The HD- Zip genes of subfamilies III and IV encode an additional conserved domain called the START (steroidogenic acute regulatory protein-related lipid transfer) domain [13] which have a putative function in sterol binding [14].

Transcription factors in HD- Zip family have been shown to take part in a diversity of developmental processes in plants and in plant adaption to environment stresses [15–17]. Over-expression of *ATHB12* results in accelerated seedling growth in *Arabidopsis* [18], *ATHB8* transcription factor directs differentiation of vascular meristems [19]. Progressive loss of the activity of *HAT3*, *ATHB4* and *ATHB2* which contained in the HD-Zip II subfamily in *Arabidopsis* causes developmental defects in embryogenesis [20]. Embryogenesis in *Arabidopsis* is also affected by HD-Zip gene activity [21, 22]. Rice *HOX12*, belongs to HD-Zip I subfamily, can modulating the expression of *EUI (ELONGATED UPPERMOST INTERNODE1)* gene and then regulates panicle exsertion [23].

In addition to the roles in plant development and growth, *HD-Zip* genes are important regulators of stress tolerance. *ATHB7* and *ATHB12* belong to HD-zip I in *Arabidopsis* and are sensitive to ABA treatment and to water deficit [24, 25]. Meanwhile, *ATHB6* has been shown to negatively regulate the ABA signaling pathway [26], while *CaHB1* and *ATHB13* show resistance to biotic stress [27, 28]. Furthermore, *SiHZ24* was been shown to modulate ascorbate, an antioxidant that scavenges reactive oxygen species (ROS), accumulation in tomato [29]. However, little is known about the HD-Zip family in grapes.

In our study, 32 *HD-Zip* transcription factors were found to be expressed in ovules of Thompson Seedless and Pinot noir grapes. A total of 21 of them were differentially expressed (Additional file 1: Table S1, unpublished data), this result conflicts with that of a previous report which states that grape has 31 *HD-Zip* transcription factors [30]. Thus, a further survey of the HD-Zip family should be conducted in the grape genome. A total of 33 putative *VvHD-Zip* genes were identified, their expression in somatic embryogenesis, different organs of Thompson Seedless, and ovules of Thompson Seedless and Pinot noir were determined, which indicate that they may take part in various process in grape development. The results provide a foundation for further functional research on *HDZ* genes in grape.

## Methods

### Plant materials

Thompson Seedless and Pinot noir grapes were grown in the germplasm vineyard of Northwest A&F University. These were managed following local standards for fertilization, irrigation and pest-management etc. Leaves, stems, tendrils, roots and flowers of Thompson Seedless were collected. Ovules were isolated from Thompson Seedless and Pinot noir in 2014 on 20 (small globular embryo in PN and TS), 30 (globular embryo in PN and TS), 40 (torpedo embryo in PN and aborted embryo in TS) and 50 (cotyledon embryo in PN and empty embryo sac in TS) days after flowering (DAF). Somatic embryos of Thompson Seedless were induced as previously described [31]. Proembryogenic masses (PEM), globular embryos (GE), heart embryos (HE), torpedo embryos (TE) and cotyledon embryos (CE) of Thompson Seedless were separated and stored at -80°C pending use (Additional file 2: Figure S1).

### Genome-wide identification and annotation of grape HD-zip genes

HD-zip domain (PF00046) was downloaded from Pfam (http://pfam.xfam.org/) and then used for identification of the HD-Zip genes from the Grape Genome Database (12 X) (http://www.genoscope.cns.fr) using HMMER3.1 [32]. Genes with default E-values (<1.0) were collected and the integrity of the HD-Zip domain was further confirmed with E-value <0.1 using the online software SMART (http://smart.embl-heidelberg.de/). Genes which contained both the conserved HD (PF00046) and LZ (PF02183) domains were preserved as HD-Zip family members. Finally, the non-redundant, confirmed genes were assigned as the family of grapevine HD-Zip genes.

### Phylogenetic, exon–intron structure and conserved motif analyses of the VvHD-zip family

MEGA 5.0 was used to construct phylogenetic trees, Neighbor-Joining (NJ) and Minimal Evolution (ME) methods were used, the bootstrap test was set as1000 iterations. Exon/intron structures of the VvHDZs were determined based on their coding sequences and their respective full-length sequences in Grape Genome Browser (http://www.genoscope.cns.fr/externe/GenomeBrowser/Vitis/), and diagrams were obtained by using online program Gene Structure Display Server (GSDS: http://gsds.cbi.pku.edu.cn). Only the exons were drawn to scale because introns of several *VvHDZ* genes were relatively too long. The MEME program (version 4.8.1, http://meme.nbcr.net/meme/cgibin/meme.cgi) was used for identification of conserved motifs (set the motif number: 20, the rest with the default settings). Discovered motifs with E-value≤1e-30 were searched in InterPro database [33].

### Chromosome localization and synteny analysis

Each grape HD-zip transcription factors (TFs) was mapped onto their corresponding chromosome at the Grape Genome Database (12 X) using the grape genome browser. Synteny blocks within the grape genome and between grape and *Arabidopsis* genomes were obtained from the Plant Genome Duplication Database (http://chibba.agtec.uga.edu/duplication), synteny blocks contained in grape and *Arabidopsis* HD-zip genes were identified. MCScanX software was employed to detection of synteny and collinearity between all possible pairs of genomes [34], BLASTP results with E-value>1e-5 were cut-off. The synteny diagrams were drawn by using the program Circos (version 0.63) (http://circos.ca/).

### Reverse-transcription quantitative PCR

Multiple ovules and embryos were pooled together to give sufficient tissue for RNA extractions. Total RNA was isolated from somatic embryos, ovules of Thompson Seedless and Pinot noir or tissue samples (roots, leaves, tendrils, stems and flowers) using an EZNA Plant RNA Kit (R6827-01, Omega Bio-tek, USA). Then, cDNA synthesis was carried out using PrimeScript RTase (TaKaRa Biotechnology, Dalian, China). Gene-specific primers for each VvHD-zip gene were designed by using Primer 6.0 (Additional file 3: Table S2). Real-time quantitative PCR was carried out as describe previously [34]. Grape (*V. vinifera*) Actin1 (AY680701) as an endogenous control, determination of the relative expression of the target gene was performed using the 2^-ΔΔc(t)^ method. All reactions were run in three biological and technical replicates for each sample. Finally, expression profiles of VvHDZ genes in different organs from the RT-PCR were collated, Least Significant Difference test (p < 0.05) was performed to analyze variance (ANOVA) using SPSS 18.0 Software (SPSS Inc., Chicago, IL). The relative expression values were log2 transformed, average linkage method provided in Cluster 3.0 was used to cluster gene and tissue types and visualized using TreeView software [35].

#### Promoter Analysis

The 1,500 bp upstream sequences of coding region of VvHDZ genes were downloaded from Grape Genome Database (12 X) (http://www.genoscope.cns.fr). The cis-regulatory elements were identified using online program PlantCARE (http://bioinformatics.psb.ugent.be/webtools/plantcare/html/) [36]. In this study, we selected cis-element associated with hormone responses, defense responses, drought responses, low temperature, heat stress and endosperm, seed-specific, shoot-specific expression and light responsive elements and meristem development.

### Subcellular localization and transactivity of the VvHDZ28 proteins

The full-length DNA of VvHDZ28 was generated from TS cDNA by using forward primer (VvHDZ28F: 5’- ATGGAGAGCAGAGGGTGTTCG - 3’) and reverse primer, VvHDZ28R: 5’- TTAACTACTCCAGAAGTCCCACAA - 3’), and cloned into *EcoR I* and *BamH I* sites, then fused in the pGBKT7 vector (PT3248-5, Clontech, USA). The VvHDZ28-pGBKT7 plasmid was transformed into Y2H Gold (630489, Clontech, USA), which carries reporter genes *AUR1C* and *MEL1*, under the control of a GAL4-responsive upstream activating sequence (UAS) and promoter elements, if the *AUR1C* and *MEL1* are activated, yeast cells can survive on SD/-Trp medium supplement with toxic drug Aureobasidin A (AbA) and turn blue in the presence of the chromagenic substrate X-a-gal. Then transformants were selected on synthetic dextrose medium lacking tryptophan (SD/-Trp) at 28°C for 2 to 3 days. Yeast transformants (pGBKT7 and VvHDZ28-pGBKT7) from SD/-Trp were then streaked onto solid SD/-Trp+AbA+X-ɑ-gal medium to score the growth response after 3 days.

The whole coding sequence of the VvHDZ28 coding regions without the termination codon were inserted into a pBI221 vector harboring the GFP protein driven by the CaMV 35S promoter by *BamH I* and *Xba I* clone site. The target vectors 35S:: VvHDZ28-GFP was used for subcellular localization. pBI22-GFP, with the free-GFP under CaMV35S was used as a positive control, WRKY33 was used as nuclear localization marker gene. Fused protein VvHDZ28-GFP and control vector 35S-GFP were transformed into protoplasts of *Arabidopsis*, and observed under a Zeiss confocal microscope (LSM510; Carl Zeiss Thornwood, NY), excitation wavelength: 488 nm, emission wavelength: 510±20 nm.

## Results

### Identification and annotation of grape HD-zip genes

The HD-Zip domain (PF00046) was download from Pfam and used for genome-wide identification of HD-Zip in grape using Hidden Markov Model (HMM) profile. Then integrity of the HD-Zip domain was determined using the online program SMART (http://smart.embl-heidelberg.de/) and sequence alignment. Finally, 33 non-redundant genes were defined as grape HD-Zip genes. These genes were named sequentially from VvHDZ1 to VvHDZ33 based on the CRIBI ID from top to bottom (Table 1), gene names in this study compared with the previous report are shown in Additional file 4: Table S3. Length of identified HD-Zip protein sequences (aa) was quite different in *V. vinifera* ranging from 171 (VvHDZ17) to 845 (VvHDZ18), with an average length of 458 aa, two extra protein (VvHDZ09 and VvHDZ17) were identified in contrast with the previous report [30]. In addition, protein length of some genes such as VvHDZ27, VvHDZ08 and VvHDZ32 are different with the previous report. CDS sequences of VvHDZ27, VvHDZ08 and VvHDZ32 were cloned from Thompson Seedless. All have the same sequence length predicted in this study (data were not shown). Usually, there were one or more *V. vinifera* HD-Zip orthologues genes in *Arabidopsis*, however, sometimes there were no *V. vinifera* orthologous HD-Zip genes in *Arabidopsis*. The detailed information of HD-Zip family genes in *V. vinifera* is listed in Table 1, including accession numbers, protein length, location and similarities to *Arabidopsis* orthologues.

**Table 1:** Detail information of Grape HD-zip genes

Gene name	Gene locus ID	Gene CRIBI ID	Accession no.	Chr	CDS (bp)	ORF (aa)	At ortholog locus	At locus description	E-value
VvHDZ01	GSVIVT01005821001	VIT_00s0299g00100	XP_002263193	chr:Un	894	297	AT4G16780.1	HAT4	1.00E-84
VvHDZ02	GSVIVT01002447001	VIT_00s0732g00010	XP_002271511	chr:Un	852	283	AT4G37790.1	HAT22	2.00E-68
VvHDZ03	GSVIVT01011754001	VIT_01s0011g04870	XP_010657445	chr:1	678	225	AT2G01430.1	ATHB17	4E-63
VvHDZ04	GSVIVT01020078001	VIT_01s0026g01550	XP_002269605	chr:1	966	321	AT3G01470.1	HAT5	7.00E-33
VvHDZ05	GSVIVT01020033001	VIT_01s0026g01950	XP_002276889	chr:1	858	285	AT1G69780.1	ATHB13	7.00E-97
VvHDZ06	GSVIVT01013073001	VIT_02s0012g02030	XP_010663102	chr2	2397	798	AT5G46880.1	HDG5	0
VvHDZ07	GSVIVT01019655001	VIT_02s0025g02590	XP_002280048	chr:2	579	192	AT3G61890.1	ATHB12	3.00E-30
VvHDZ08	GSVIVT01035612001	VIT_04s0008g03250	XP_002283717	chr:4	2523	840	AT1G52150.1	CNA	0
VvHDZ09	GSVIVT01019012001	VIT_04s0023g01330	XP_002273007	chr:4	636	211	AT4G36740.1	ATHB40	3.00E-44
VvHDZ10	GSVIVT01035238001	VIT_04s0079g00480	XP_002268272	chr:4	2145	714	AT1G73360.1	HDG11	0
VvHDZ11	GSVIVT01025193001	VIT_06s0004g02800	XP_010651163	chr:6	2535	844	AT5G60690.1	REV	0
VvHDZ12	GSVIVT01003431001	VIT_07s0191g00180	XP_003632476	chr:7	1008	335	AT2G22430.1	ATHB6	2.00E-58
VvHDZ13	GSVIVT01033744001	VIT_08s0007g04200	XP_002283931	chr:8	792	263	AT5G03790.1	ATHB51	7.00E-41
VvHDZ14	GSVIVT01033481001	VIT_08s0007g06670	XP_002275747	chr:8	996	331	AT5G06710.1	HAT14	2.00E-66
VvHDZ15	GSVIVT01017010001	VIT_09s0002g03740	XP_002284003	chr:9	2517	838	AT1G52150.1	ATHB15	0
VvHDZ16	GSVIVT01017073001	VIT_09s0002g04340	XP_002284502	chr:9	2265	754	AT4G16780.1	HAT4	2.00E-56
VvHDZ17	-	VIT_10s0003g00380	XP_002273463	chr:10	516	171	AT5G53980.1	ATHB52	3.00E-24
VvHDZ18	GSVIVT01021625001	VIT_10s0003g04670	XP_002281868	chr:10	2538	845	AT2G34710.1	PHB	0
VvHDZ19	GSVIVT01012643001	VIT_10s0116g00680	XP_002266688	chr:10	2181	726	AT4G21750.1	ATML1	0
VvHDZ20	GSVIVT01030605001	VIT_12s0059g02310	XP_010657311	chr:12	2274	757	AT1G05230.3	HDG2	0
VvHDZ21	GSVIVT01016272001	VIT_13s0019g04320	XP_002274194	chr:13	2523	841	AT5G60690.1	REV	0
VvHDZ22	GSVIVT01001366001	VIT_13s0156g00260	XP_002268178	chr:13	1077	358	AT5G06710.1	HAT14	8E-59
VvHDZ23	GSVIVT01032491001	VIT_14s0066g01440	XP_002278872	chr14	822	273	AT3G01470.1	HAT5	3.00E-72
VvHDZ24	GSVIVT01011377001	VIT_14s0108g00390	XP_010661046	chr:14	867	288	AT1G69780.1	ATHB13	1.00E-68
VvHDZ25	GSVIVT01018247001	VIT_15s0021g01880	XP_010661380	chr:15	858	285	AT4G16780.1	ATHB4	2.00E-56
VvHDZ26	GSVIVT01027508001	VIT_15s0048g02000	XP_010661562	chr:15	2433	810	AT4G00730.1	ANL2	0
VvHDZ27	GSVIVT01027407001	VIT_15s0048g02870	XP_002262950	chr:15	747	248	AT2G46680.1	ATHB7	1E-54
VvHDZ28	GSVIVT01038619001	VIT_16s0098g01170	XP_002271523	chr16	681	226	AT3G61890.1	ATHB12	3E-36
VvHDZ29	GSVIVT01010600001	VIT_16s0100g00670	XP_010662507	chr:16	2352	783	AT4G00730.1	ANL2	0
VvHDZ30	GSVIVT01008065001	VIT_17s0000g05630	XP_002271692	chr:17	954	317	AT3G01470.1	ATHB1	4.00E-37
VvHDZ31	GSVIVT01029396001	VIT_17s0053g00780	XP_002271012	chr:17	2148	715	AT1G73360.1	ATHDG11	0
VvHDZ32	GSVIVT01009083001	VIT_18s0001g06430	XP_002285743	chr:18	864	287	AT4G40060.1	ATHB16	8.00E-47
VvHDZ33	GSVIVT01009274001	VIT_18s0001g08410	XP_002283547	chr:18	813	270	AT4G37790.1	HAT22	1E-59

*Chr* Chromosome, *CDS* coding sequence, *ORF* open reading frame

### Phylogenetic analysis, conserved structural features of the grapevine HD-zip gene family

**Fig. 1:**
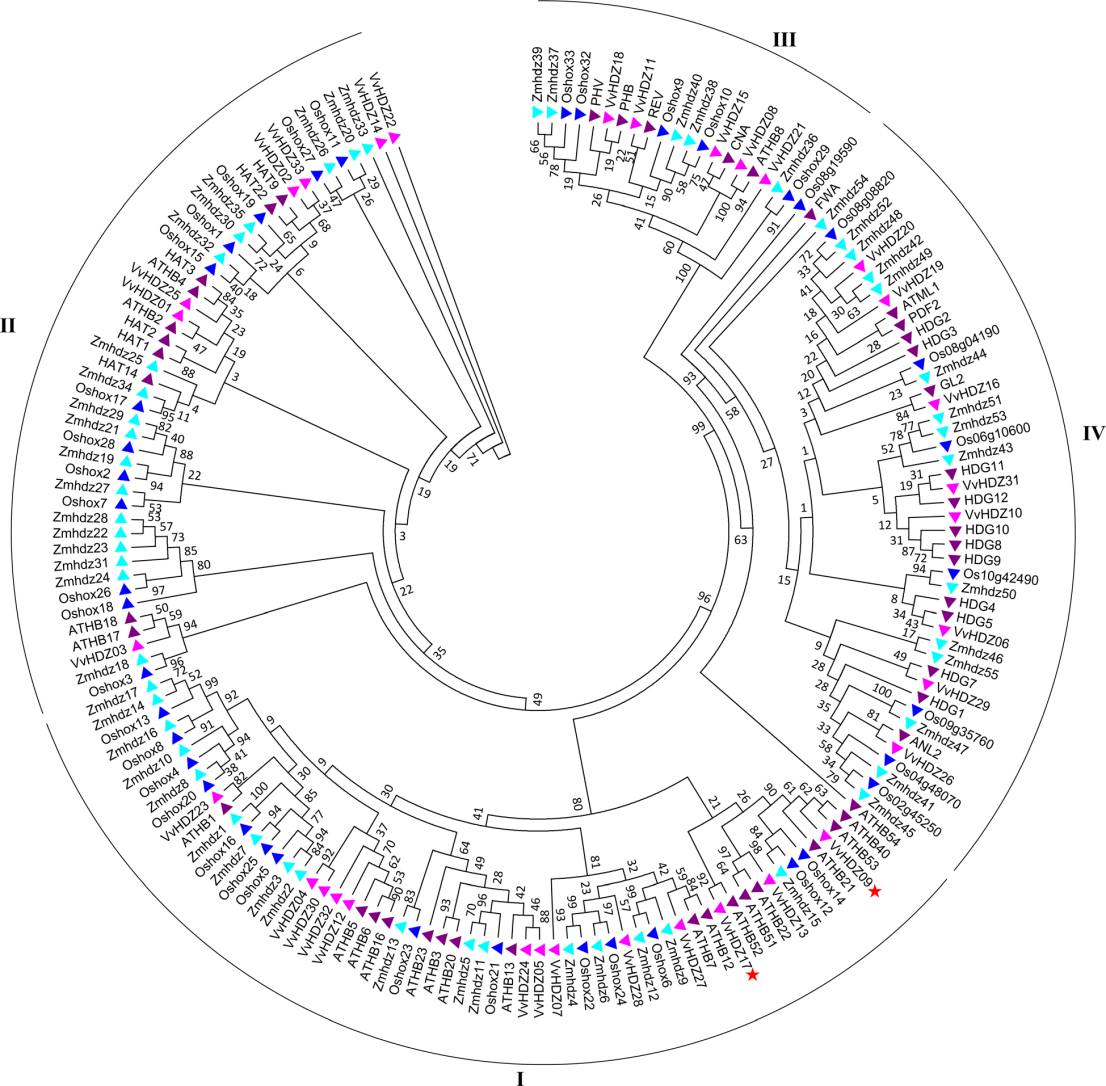
The phylogenetic tree of grape *HD-zip* genes. Members of the *HD-zip* genes from grapevine, *Arabidopsis*, maize and rice are marked: pink, purple, blue and turquoise, respectively. The phylogenetic tree was generated by MEGA 5.0 using the Neighbor-Joining method, bootstrap test (1000 replicates), two new identified genes were labeled by red star

**Fig. 2:**
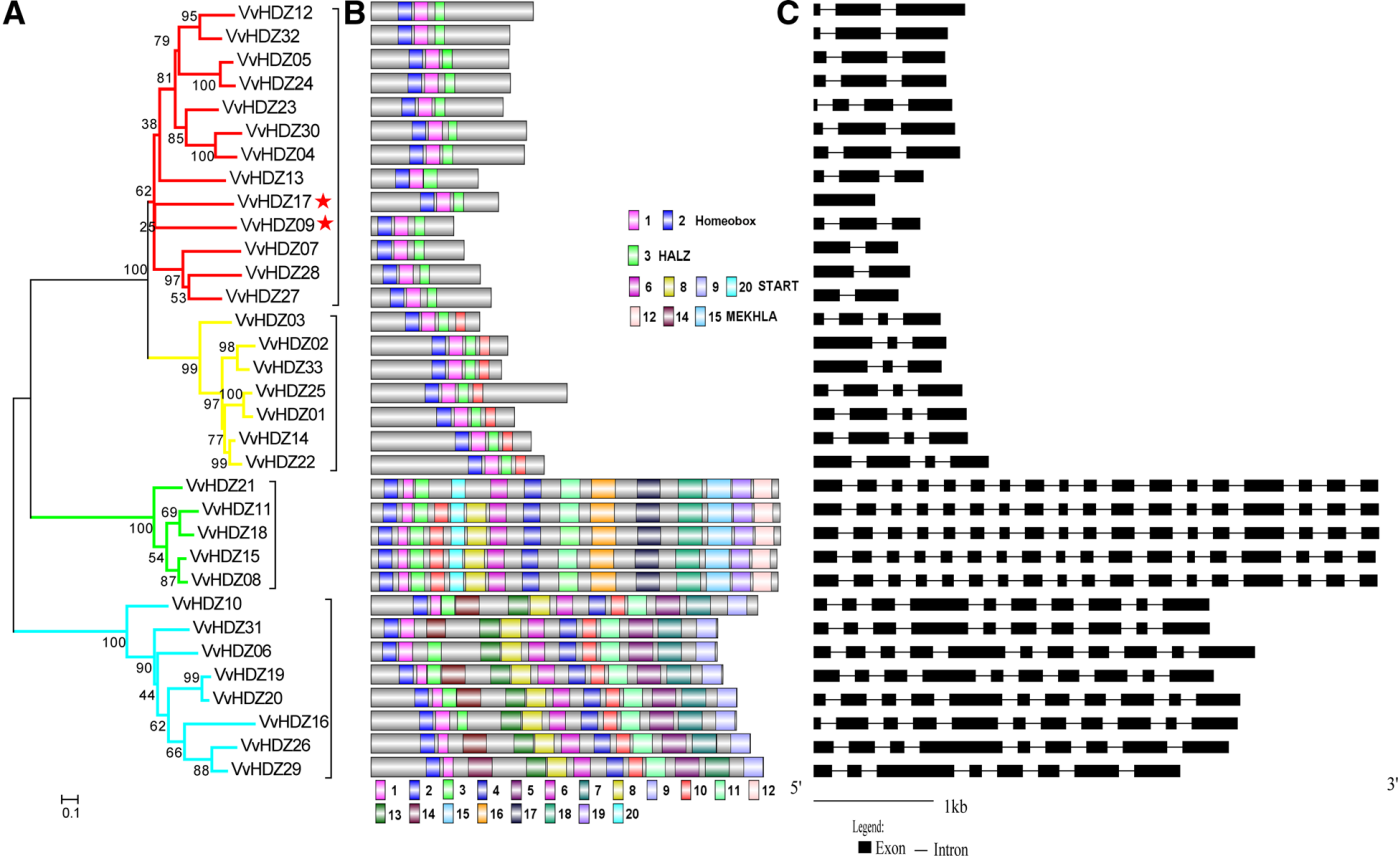
Structure characteristics of the HD-zip family transcription factors in grape. **a** Phylogenetic analysis of VvHD-zip proteins, genes in subfamilies I-IV are marked with red, green, yellow and turquoise lines, respectively, two new identified genes were labeled by red star; **b** MEME analysis of protein motifs in grape; **c** Exon and intron structure analysis of VvHD-zip transcription factors

To illustrate the phylogenetic relationship of the *HD-Zip* gene families in grape and other species, protein sequences of the HD-Zip, 33 from grapevine (*V. vinifera* L.), 48 from *Arabidopsis* (*Arabidopsis thaliana*), 55 from maize and 48 from rice (Additional file 5: Text S1) [11, 12, 20, 37–39] were used to generate a phylogenetic tree. The HD-Zips in grape can be classified into four subfamilies (Figs. 1 and 2a) based on the phylogenetic tree, there are 13, 7, 5 and 8 members in the four HD-Zip subfamilies I, II, III, IV, respectively. Classification of HD-Zip family is consistent with previous report [30] except two new identified genes belong to HD-Zip I subfamily. The number of each subfamily is differed from *Arabidopsis*, maize and rice (Table 2)*.* Previous reports showed that the HD-Zip III subfamily is highly conserved in land plants [12]. The same number of HD-Zip III genes have been identified in this study, *Arabidopsis* and maize [12, 40].

**Table 2:** Numbers of HD-Zip genes in the grape, *Arabidopsis*, maize and rice genomes

Species	Grape	Arabidopsis	Rice	Maize
Class I	13	17	14	17
Class II	7	10	13	18
Class III	5	5	9	5
Class IV	8	16	12	15
Total number	33	48	48	55

We identified 20 conserved protein domains with E-value ≤1e-30 (Additional file 6: Figure S2) in *V. vinifera* HD-Zip using the online MEME tool (Fig. 2b), motifs 1 and 2 in the N-terminal region of the protein are conserved in 33 members in the HD-Zip family. Members in HD-Zip I and II subfamily have the same numbers and protein domains. In addition, all members in HD-Zip III subfamily have MEKHLA domain in their C-terminal region, moreover, genes in HD-Zip III and IV have START domains, consistent with *Arabidopsis* and maize [38, 39]. We noticed that motifs have similar orders in the same subfamily. With some exceptions, most HDZ proteins have the same motifs in contrast with previous report [30]. For example, in HD-Zip I subfamily, the Vvhdz3 (VvHDZ12 in this study) have 14 conserved motifs like protein in HD-Zip III subfamily.

Exon/intron structures was reported to play pivotal roles during the evolution of multiple gene families [41, 42]. In grape, structures of the *HD-Zip* genes were obtained by analysing boundaries of exon/intron. Similar to previous reports for *Arabidopsis* and rice, the numbers of introns and exons are quite diffed in four subfamilies. As shown in Fig. 2c, genes in HD-Zip I and II have 2-4/3-4 exon/intron, except *VvHDZ13* which has only one exon. Genes in HD-Zip III have 18/17 exon/intron, while genes in HD-Zip IV have 8/7 or 11/10 exon/intron. Most intron/exon structures of the HDZ gene in this study are the same as in the previous publication [30], though some are different, including *VvHDZ02*, *VvHDZ05*, *VvHDZ12* and *VvHDZ32*. We note that *HDZ* genes in the same subfamily (II, III and IV) have similar numbers of exon/intron, and that the exon–intron structures of the HD-Zip genes are similar across species [12, 39, 40, 43]. More divergences were found in HD-Zip I, exon/intron is 1/0, 2/1, 3/2 or 4/3. The results suggest that the HD-Zip family are conserved in plant evolution.

### Synteny analysis of HD-zip genes

**Fig. 3:**
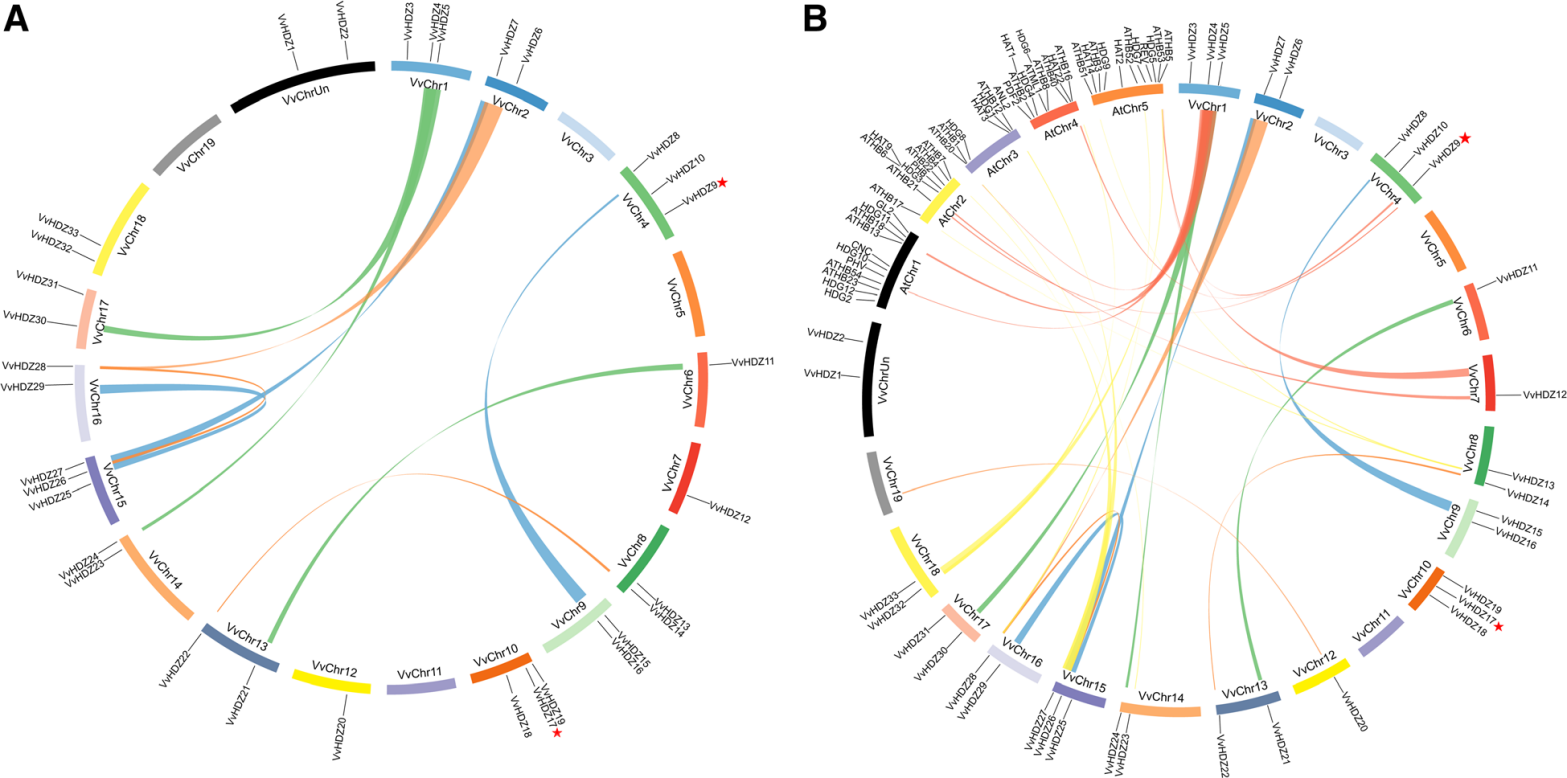
Synteny analysis of *Vitis vinifera* and *Arabidopsis HD-zip* genes. **a** Synteny analysis of *V. vinifera HD-zip* genes. Chromosomes 1-19 are shown in a circular form. The approximate distribution of each VvHDZ gene is marked with a short black line on the circle. Colored curves denote the details of syntenic regions between the grape HD-zip genes. **b** Synteny analysis of HD-zip genes bewteen *V. vinifera* and *Arabidopsis*. The *V. vinifera* and *Arabidopsis* chromosomes are drawn as circles. Location of each *AtHB* and *VvHDZ* gene is marked with a short black line on the circle. The colored curves denote the syntenic regions of the *V. vinifera* and *Arabidopsis HDZs* genes. Two new identified genes were labeled by red star

Genomic comparison is a rapid method for transferring genomic information from a model species to a less-studied species [44, 45]. In grape, 33 *HD-Zip* genes located on the 16 chromosomes (Fig. 3a), two new identified protein, VvHDZ09 and VvHDZ17 located on chromosome 4 and 10, and the other genes have same locations with the previous report [30]. Each chromosome has one or more *HD-Zip* genes, except for chromosomes 3, 5 and 11. Tandem duplication events do not occur in grape according to the method of Holub [46]. However, 9 segregation duplication events with E-value<1e-5 were identified (Fig. 3a, Additional file 7: Table S4), indicating that some *HD-Zip* genes were possibly generated by gene duplication.

*HD-Zip* genes in *Arabidopsis* have been widely investigated [18–20, 47], therefore, a synteny analysis between *Arabidopsis* and grape *HD-Zip* genes was carried out to determine whether this might provide some functional insights (Fig. 3b and Additional file 8: Table S5). The synteny analysis of *V. vinifera* and *Arabidopsis HD-Zip* revealed a total of 16 pairs of syntenic *HD-Zip* genes with E-value<1e-5 between *V. vinifera* and *Arabidopsis*, including eight *VvHD-Zip* genes and 14 *AtHD-Zip* genes, respectively (Fig. 3b, Additional file 8: Table S5). This indicates most of the *HD-Zip* genes arose before the divergence of *Vitis* and *Arabidopsis*.

### Expression profiles of HD-zips in somatic embryo and ovules of seedless and seeded grapes

**Fig. 4:**
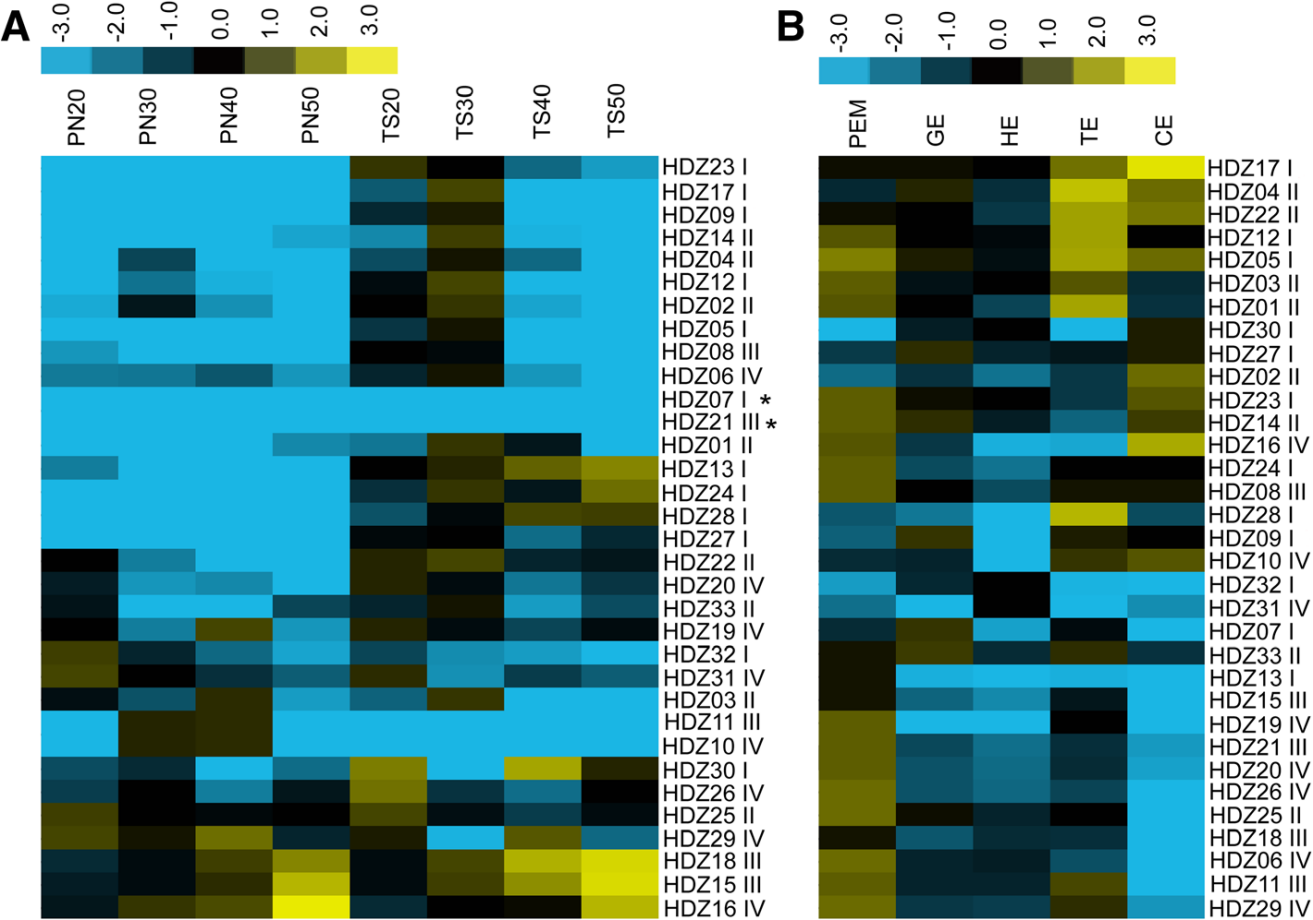
Expression analysis of HD-zip family genes in different organs in *Vitis vinifera*. Transcript levels of the HD-Zip gene family in ovules of Pinot Noir and Thompson Seedless (**a**) and somatic embryo of Thompson Seedless (**b**). The colour scale up the heat map represent expression values; blue represent low transcript abundance while yellow represent high level of transcript abundance. Genes with no significant differences in all stages were labeled by black asterisk. The relative expression values were log2 transformed, the heat map was generated using cluster 3.0 software and visualized using TreeView software

The HD-Zip genes have been shown to regulate embryo develop in *Arabidopsis* [12, 20] and reproductive progress in rice and barley [22, 23]. To determine the potential roles of *HD-Zips* in grape ovule abortion or ovule development, the distribution of the 33 *HD-Zips* gene transcripts were surveyed in the ovules of TS (seedless) and PN (seeded) at 20, 30, 40 and 50 DAF (embryo aborted at 30 to 40 DAF, Fig. 4). Most genes in HD-zip I and II have high transcript levels in TS30, while genes in HD-zip IV expressed highly in TS20. We noticed that most genes enriched in TS were poorly expressed in PN, and *vice versa* (Fig. 4a and Additional file 9: Figure S3), for example, *VvHDZ28* in HD-zip I and *VvHDZ11* in HD-zip III. Two out of 33 genes, *VvHDZ07* and *VvHDZ21* were not detected in either PN or TS which indicates that they did not take part in ovule development in either PN or TS.

To further analyze the relationship between *HD-Zips* during embryogenesis, expression levels were detected in somatic embryos of TS at the stages of PEM, GE, HE, TE and CE (Fig. 4b and Additional file 10: Figure S4). A total of six HD-Zip I genes (*HDZ04, 05, 12, 17, 23* and *24*) have high transcript levels during somatic embryogenesis; all HD-zip II members except *VvHDZ02* have high transcript levels in PEM and TE; four out of five genes in HD-zip III present lower transcript levels in CE; HD-Zip IV genes have more dynamic expression patterns in somatic embryogenesis. Most of these have higher expressions in PEM and lower expressions in CE. *VvHDZ07* and *VvHDZ21* were not expressed in PN and TS but were expressed in PEM, GE and TE. The results suggest that *VvHDZ* genes in grape take part in embryogenesis of somatic embryos and zygotic embryos in grapes.

### Expression profiles of HD-zips and different organs in TS

**Fig. 5:**
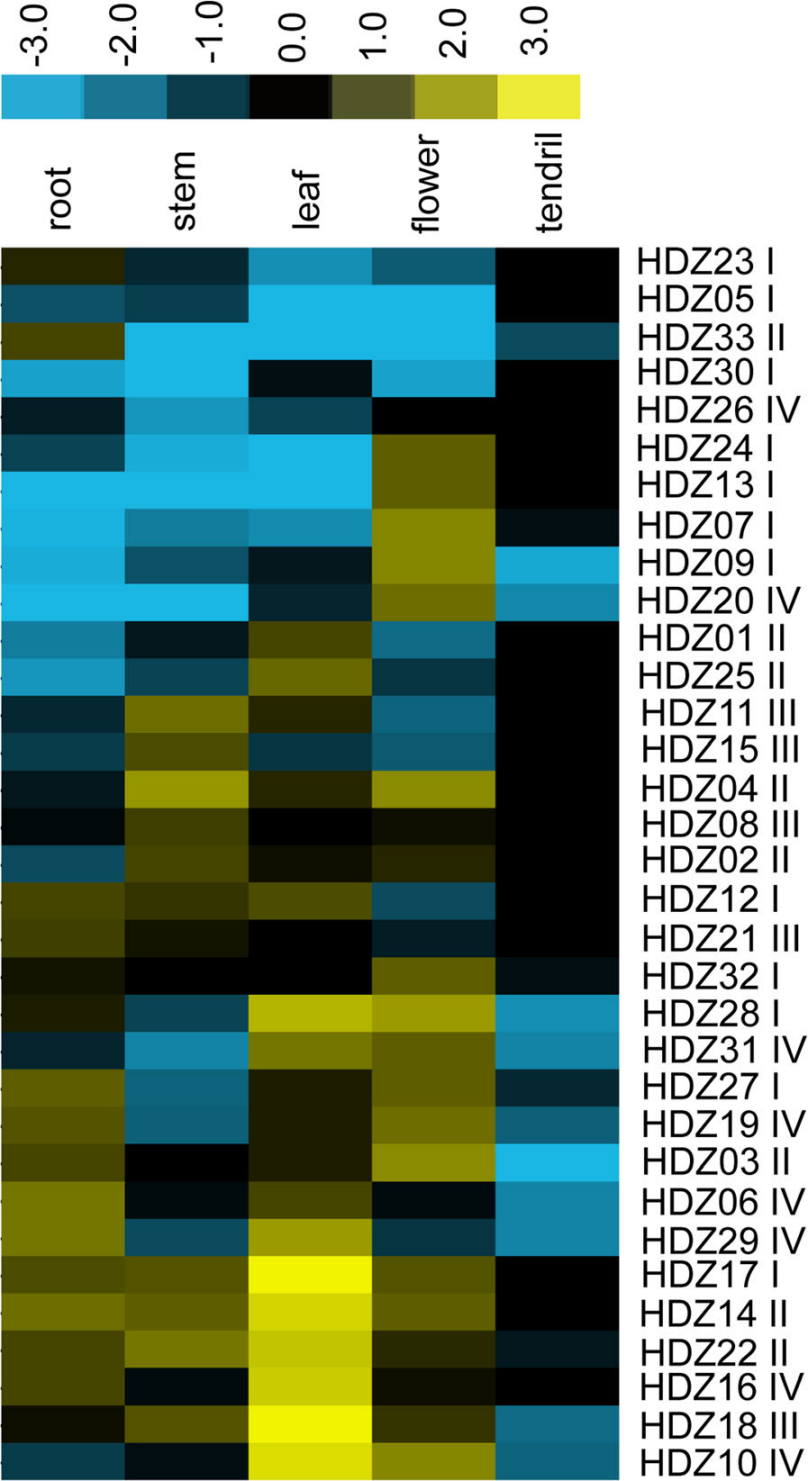
Expression pattern of the grape HD-zip family in different organs of Thompson Seedless. The experiments were repeated three times. The colour scale up the heat map represent expression values; blue represent low transcript abundance while yellow represent high level of transcript abundance. The relative expression values were log2 transformed, the heat map was generated using cluster 3.0 software and visualized using TreeView software

To further investigate the expression of HD-Zip in grape development, we examined the expression of *HD-Zip* in shoots, stems, leaves, flowers and tendrils. All the grape *HD-Zip* genes were expressed in the various tissues at some level or another. Based on the expression profiles, nearly half of the *VvHDZs* were expressed in flowers and leaves, no tissue-specific genes were found. However, some clear spatial differences were noted (Fig. 5 and Additional file 11: Figure S5). For instance, *HDZ24* and *HDZ13* have higher expression in flowers, while *HDZ24*, *HDZ22* and *HDZ01* had high transcript levels in leaves. The HD-zip genes which showed no significant transcription differences among different tissues are likely to play a more extensive role during grapevine development.

### Cis-elements analysis in the promoter region of grape HD-zip genes

**Fig. 6:**
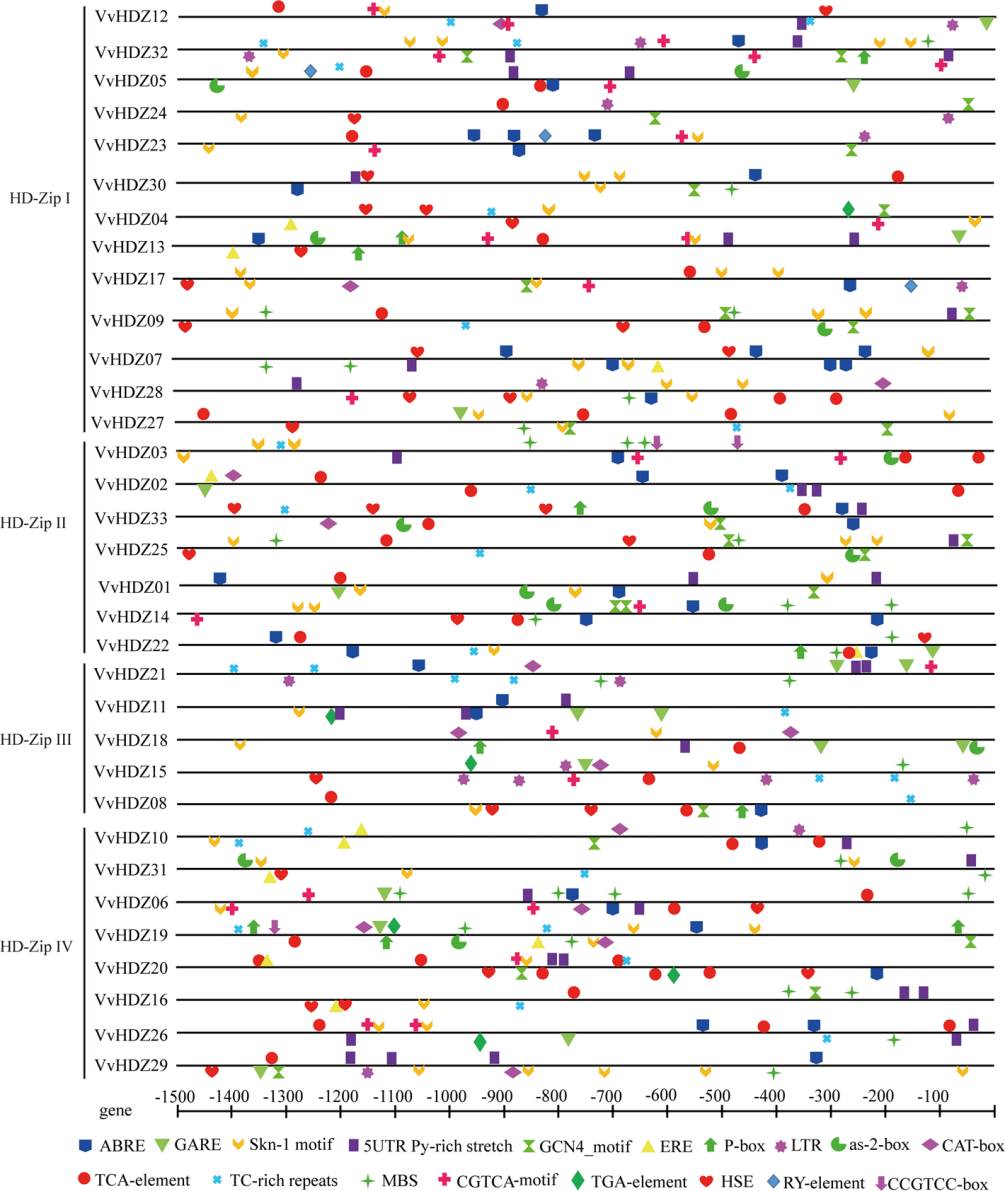
Promoter cis-element analysis of *VvHDZ* genes. 1.5 kb upstream promoter sequence for all *VvHDZ* genes was downloaded from the grape database, number and position of various cis-acting regulatory elements were scanned through PlantCARE. Different regulatory elements are represent by different colored symbols and placed in their relative positions on the promoter. Symbols presented above the line indicate the forward strand of DNA, while those below indicate the reverse strand

To access stress responsive expressions of VvHDZ genes following hormone or defense treatments, the upstream 1500bp promoter sequence for each VvHDZ gene was retrieved from grape and analyzed for the presence of cis-acting elements using PlantCARE (Fig. 6). We identified several hormone-responsive cis elements such as ABRE, GARE, TCA, CGTCA box and TGACG motif and stress responsive elements such as: LTR, MBS, Tc-rich repeats, element conferring high transcription level (5’ UTR Py-rich stretch). Most genes have at least one endosperm expression element except HDZ02 and 21. Some have seed–specific regulation binding site (RY-element), genes containing the skn-1 motif have expression in ovules of PN and TS and different levels except *VvHDZ07*. *VvHDZ09* and *VvHDZ25* which have similar cis-elements. All these motifs play important roles in regulating the expressions of various stress responsive genes. In addition, all these motifs were found to be distributed apparently randomly in both the positive and negative strands of promoter sequences.

### VvHDZ28 locates to the cellular nucleus and shows transcriptional activity

**Fig. 7:**
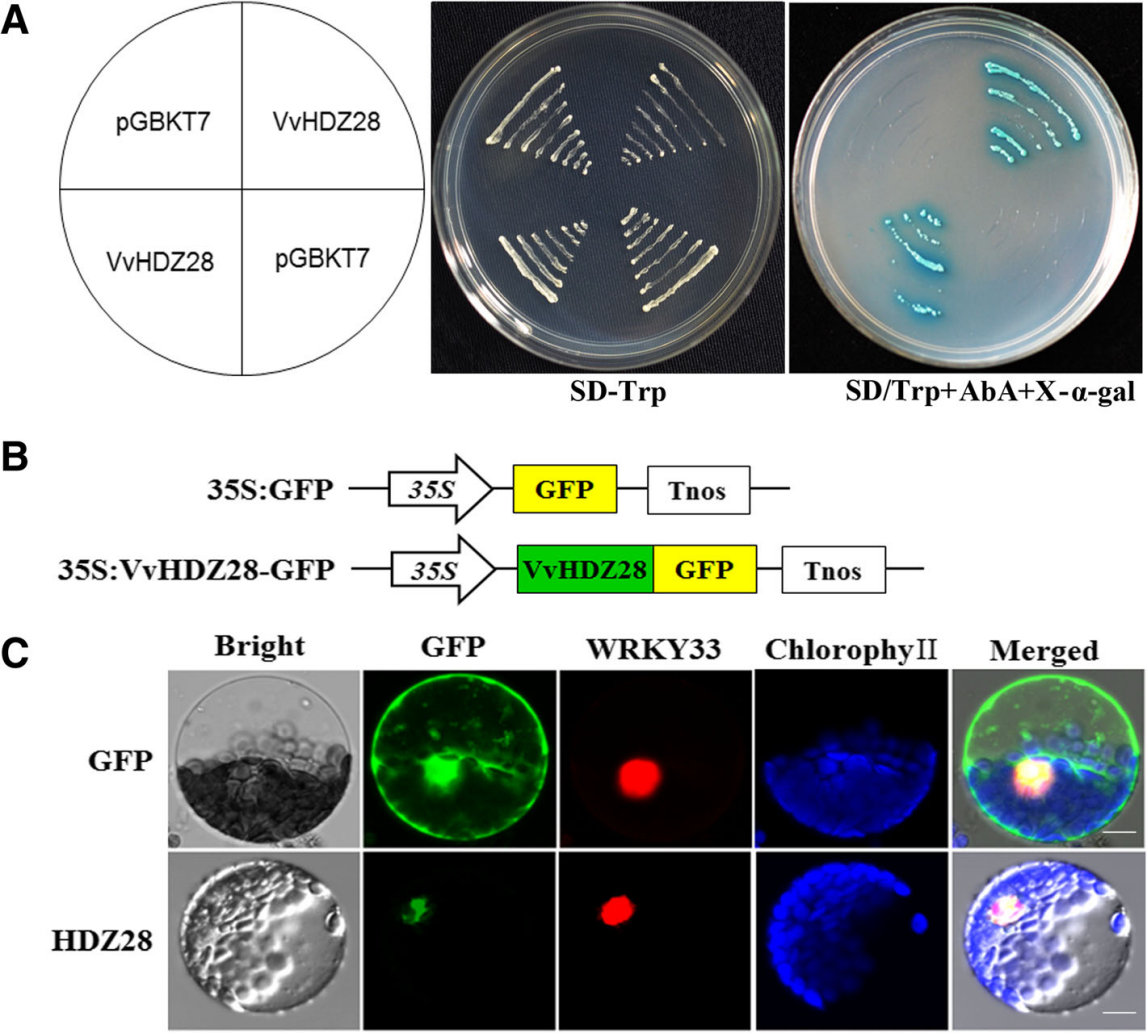
Subcellular localization and transcriptional activity of VvHDZ28. **a** Growth of yeast cells transformed with pGBKT7/VvHDZ28, using pGBKT7 as a control. **b** Schematic diagrams of the constructs used for the subcellular localization assay. **c** Subcellular localization of VvHDZ28 in *Arabidopsis* leaf protoplasts. 35S-GFP was used as positive control, WRKY33 (At2g38470) was used as nuclear localization marker gene. Results shown are representative of three independent experiments (n =3). Bars, 200 μm

The full length of VvHDZ28 was isolated from TS, containing an ORF of 678 bp, encoding 225 amino acids, it contain HD domain and downstream LZ domain (Additional file 12: Figure S6). A yeast GAL4 system was used to determine the transcription activity of VvHDZ28. Fusion plasmid pGBKT7 - VvHDZ28 was transformed into the yeast strain Y2H; the pGBKT7 vector was employed as a negative control. Yeast cells transformed with the pGBKT7 control vector or pGBKT7 - VvHDZ28 grew well on (SD/-Trp). However, the yeast cells transformed with the control vector did not survive on selective synthetic dextrose medium lacking tryptophan and supplement with Aba (Aureobasidin A) and X-ɑ-gal (SD/-Trp+AbA+X-ɑ-gal), while strong blue signals could be seen in yeast transformed with pGBKT7 - VvHDZ28, suggesting that the VvHDZ28 protein has transcriptional activity in yeast (Fig. 7a).

To investigate whether VvHDZ28-GFP proteins located on nucleus, fused construct 35S::VvHDZ28-GFP (Fig. 7b) was transiently transformed into *Arabidopsis* protoplasts. The 35S::GFP construct was used as the positive control and WRKY33 as nucleus marker [46]. VvHDZ28-GFP was restricted within the nucleus of *Arabidopsis* protoplasts and overlapped with WRKY33 while the control GFP fusion protein was targeted both the nucleus and the cytoplasm. These results demonstrate that VvHDZ28 is nuclear protein, and function as a transcription factors (Fig. 7c).

## Discussion

In both animals and plants, the basic body plan is laid down during embryogenesis. Embryogenesis is completed in seeded grapes whereas with stenospermocarpy embryo abortion occurs. In the last decade, molecular genetic studies have uncovered a large number of regulatory genes involved in plant development, including homeodomain-leucine zipper (HD-Zip) family [17, 22, 48, 49]. In our study, 21 HD-Zip genes were differentially expressed in ovules of seeded and seedless grapes (Additional file 1: Table S1, unpublished data). Also, HD-Zip gene family has been widely studied in both monocots and dicots [20, 22, 23, 27, 29, 48]. However, their functions remain obscure in grapes. This study reveals the potential role of the HD-Zip genes in various aspects of grape development.

### Identification of HD-zip genes in grape

Our survey for HD-zip genes in grape was conducted to access their functions, particularly with respect to embryo abortion. In the end, 33 HD-Zip transcription factors were identified based on the 12 X grape genome (*V. vinifera* L.), in which *VvHDZ09* and *VvHDZ17* are new identified genes in our study, and both of them belonging to the HD-Zip I subfamily (Fig. 2a). This number is less than in *Arabidopsis*, rice, maize or poplar [38–40]. All were expressed during somatic embryogenesis (Fig. 4b) and in the organs: roots, stems, leaves, tendrils or flowers (Fig. 5) but at different levels. Moreover, 31 of them were detected in ovules of TS and PN grapes which suggests all the 33 VvHD-Zip genes identified are also putative HD-Zip genes.

### The evolutionary relationship of VvHD-zip genes

The VvHDZ family can be grouped into four subfamilies (I - IV) according to their relatedness with homologous HD-Zip transcription factors in other species, such as in *Arabidopsis*, maize and rice (Fig. 1) [7]. Our results are consistent with earlier reports [38, 40]. The HD-Zip III subfamily has the least number among them in our study (Fig. 2a), which is consistent with previous reports that HD-Zip III is the most conserved subfamily among various species [39, 40]. Meanwhile, the HD-Zip II and IV subfamilies occur in different numbers in different species. This is the main reason that the HD-Zip family has different numbers in various species [38, 40, 43].

Analysis also suggests that grape HDZ-Zip genes encode proteins containing conserved domains in each subfamily (Fig. 2b), motif HD and LZ were conserved in all HD-Zip genes. Each domain has specific function, the HD and LZ domains in HD-Zip genes have been reported to be responsible for protein-DNA and for protein-protein interactions, respectively [11]. The HD-Zip I target CAAT(A/T)ATTG sequence, and HD-Zip II proteins interact with similar pseudopalindromic binding sites CAAT(C/G)ATTG, slightly different sequences are recognized by HD-Zip III and IV proteins, (GTAAT(G/C)ATTAC) and (TAAATG(C/T)A), respectively [11, 22, 23]. Precise regulatory roles of the START domains have yet to be established [7]. START was shown to be required for transactivation and to interact with lipid and steroid ligands [50]. However, exact interaction mechanisms remain open to question, suggesting further research is required.

Alterations in exon–intron structure within the coding region of a gene cause changes in their function [42]. Genes in each HD-Zip subfamily have similar numbers and positions of exon–intron structure (Fig. 2c). However, more divergences were found in HD-Zip I (Fig. 2c), which indicates genes may have different functions in grape development.

Most HDZ proteins have the same motifs and intron/exon structures compared with previous report [30] with some exception. For example, motifs of Vvhdz3 (VvHDZ12 in this study) in HD-Zip I and intron/exon structures of *VvHDZ02*, *VvHDZ05*, *VvHDZ12* and *VvHDZ32*. With the updating of grape genome, some introns which defined as intron were proved to be exon, for example, *VvHDZ32* (Vvhdz6 in [30]) was clone in our experiment, and the third intron in previous report was actually exon region, and the third intron in previous report was actually exon region, its translation stop at fourth intron in previous report, only 2 intron is existed, and maybe this is the main reason for protein length and intron/exon structure differences in current study compared with reference [30].

Segregation duplication is defined as duplicated genes but presented on different chromosomes [51]. The large number of gene duplication events for grape (Fig. 4a and b) will help aid future analyses of gene function prediction and evolution. In angiosperms, whole genome duplication events are a common phenomenon [52] and often result in gene family expansion [39]. Gene duplication contributes to the evolution of novel gene functions in plants. Segregation duplication events and syntenic relations between grape and *Arabidopsis* indicates that some *VvHDZ* genes were generated by gene duplication and have the same origin.

### Various roles of HD-zip during plant development

Gene expression patterns are usually closely related to function. In our study, the expression profiles of each VvHDZ gene were investigated in somatic embryos of TS and ovules of PN and TS as well as different organs in TS (leaves, flowers, tendrils, roots and shoots). Genes in HD-Zip family were proved to involve in embryo development [12, 20, 53, 54]. Single mutant of HD-Zip I class genes do not induce any embryonic defect, but over-expression of *ATHB5* can rescue rootless phenotype of bdl (a gene mediate auxin response in embryo) [54]. *VvHDZ12* is homologous gene of *ATHB5*, it had higher expression in 20 and 30 DAF in TS. *hat3 athb4 athb2* (HD-Zip II) mutants have developmental defects in embryogenesis [20]. Their homologous genes, *VvHDZ01* mainly expressed in TS while *VvHDZ25* has no differences in PN and TS. Single mutants of HD-Zip III members do not show any defect while triple mutants of *rev phb phv* and *rev phb cna* result in globular embryo defects [12, 55], however, *rev* does display various defects post-embryonically[56]. Its homologous gene *VvHDZ11* has lower expression in TS, it may indicate that *VvHDZ11* may have potential function in embryo abortion. Double mutants of HD-Zip IV gene *atml1-3 pdf2* lead to embryonic arrest at the globular stage [53], but their homologous gene, *VvHDZ19* and *VvHDZ20* showed higher expression in TS at most stages. Considering that embryo aborted at 40 DAF in TS while it developed normally in PN, we speculated that *VvHDZ11* in HD-Zip III, *VvHDZ10* in HD-Zip IV, which were preferentially expressed in PN ovules during development (Fig. 4a and Additional file 9: Figure S3), may be regulating ovule development in surviving seeds in PN. *VvHDZ05*, *VvHDZ09*, *VvHDZ13*, *VvHDZ17*, *VvHDZ23*, *VvHDZ24*, *VvHDZ27* and *VvHDZ28* in HD-Zip I, *VvHDZ01* in HD-Zip II, which mainly expressed in TS (Fig. 4a and Additional file 9: Figure S3) may be associated with embryo abortion.

HD-zip genes involved in somatic embryo development [43, 57, 58], most grape HD-Zip I and II genes have higher expression in PEM and CE, otherwise, most genes in grape HD-Zip III and IV have higher transcript levels in PEM (Fig. 4b and Additional file 10: Figure S4). Only *VvHDZ08* had lower expression in HE, this is different from previous report that genes in HD-Zip III have higher expressions in somatic embryos at the mature stage in *Larix leptolepis* [58]. Considering that embryos aborted as ovules develop in TS while they continue to develop in PN, *VvHDZ10* and *VvHDZ11* preferentially expressed in PN30 (globular embryo) and PN40 (torpedo embryo), they were also expressed in somatic embryo at GE and TE stages, we proposed that their expression at GE and TE stages are necessary for embryo development.

Genes in the HD-Zip family also involved in different organ development. In concerning of expression HD-Zip gene in different organs in TS, HD-Zip I subfamily genes may be involved in flowers and leaves as already shown that they regulate cotyledon, spike and leaf development [15, 18, 22, 23]. For HD-Zip II, most of them have higher expressions in flowers and leaves, while genes in HD-Zip II have been shown to take part in in carpel margin, flower development [59, 60] and leaf polarity [61]. HD-Zip III in grape may have potential functions in organ polarity, vascular development, and meristem function as suggested in previous reports, because most of them have higher expressions in stems and leaves [12, 21], HD-Zip IV most of genes have higher expression in roots, leaves and flowers as the publications that HD-Zip IV modulate trichome and anther development [53, 62]. These results suggest the *VvHDZ* genes may play a variety of roles in grape development.

Promoter cis-element analysis revealed that 31 out of 33 members have the endosperm regulation motif. HD-Zip family genes have been proposed to be involved in abscisic acid (ABA)-related responses, water deficit and salt stress [11, 48, 63, 64], ABRE responsive element was founded in most members in HD-Zip I. HD-Zip II was reported to influenced by auxin [65] and drought stress [66], however, no auxin-element was found in the grape HD-Zip II subfamily while the drought stress responsive element-MBS was found in about half of them. On the other hand, the salicylic acid responsive motif TCA-element was identified in all genes in in HD-Zip II, which suggests that HD-Zip II genes expression may influenced by salicylic acid. No LTR motif was found in HD-Zip II genes compared with the other subfamilies, indicating that this subfamily may not respond to low temperature environments. According to previous publications, *ATHB8* in HD-Zip III family is affected by auxin [12, 67], *VvHDZ11* and *VvHDZ15* have auxin responsive element in our study. In addition, gibberellin may has an effect on HD-Zip III gene expression as gibberellin responsive element P-box or GARE motif was founded in all HD-Zip III genes. HD-Zip IV is responsive to more than one hormones (including ABA, SA, GA and JA) [68], water and salinity stress [43]. Genes in HD-Zip IV have defense and stress responsive element MBS or TC-rich repeats, compared with the other subfamily, HD-Zip IV genes may be affected by ethylene as all of them have at least one ERE element except *VvHDZ06*. These results suggest that HD-Zip IV has a potential role during defense environment and is influenced by ethylene.

ATHB12 regulates leaf growth by promoting cell expansion and endoreduplication [18] and the homologous gene, *VvHDZ28* has high transcript levels in flowers and leaves. *VvHDZ28* is homologous to *ATHB12*, and this gene has been shown to participate in various aspects of development in *Arabidopsis* [18, 47, 48, 69]. Its role in grape is not yet fully characterized. Here, we found that VvHDZ28 possessed the features typical of the HD-Zip family, including having transcriptional activity (Fig. 7a), and being located in the nucleus (Fig. 7c). These suggest it functions in grape as a transcription factor.

## Conclusions

We have identified 33 HD-Zip transcription factors, all members contain one or more Homeobox domains. Grape HD-Zip family can be grouped into four subfamilies, genes in each subfamily have similar exon/intron structure and motifs. The *VvHD-Zip* genes were differentially expressed in the ovules of seedless (TS) and seeded grapes (PN), their transcripts were also detected in somatic embryogenesis in TS. Furthermore, the *HD-Zip* genes were also detected in vegetative organs of TS, which indicates that, in *V. vinifera*, they have potential functions during embryo abortion and also during organ development. Moreover, *VvHD-Zip* genes have hormone response elements and endosperm expression elements as well as seed specific regulation elements. VvHDZ28 is located in the nucleus and has transcriptional activity in yeast cells. Our research not only added two new members to the grape HD-Zip family, but also provided information for further function analyses of *VvHDZ* genes.

## Additional files

Additional file 1: **Table S1.** Differential expressed HD-Zip genes in ovules transcriptome data of PN and TS in 2013 and 2014. (XLSX 13 kb)

Additional file 2: **Figure S1.** Somatic embryo of Thompson Seedless at different stages. A: proembryogenic masses, PEM; B: globular embryo, GE; C: heart embryo, HE; D: torpedo embryo, TE; E: cotyledon embryo. (TIFF 2316 kb)

Additional file 3: **Table S2.** Primers used in expression analysis of HD-zip gene family in grape. (XLSX 11 kb)

Additional file 4: **Table S3.** Comparison of HD-Zip genes names in this study and in reference [30]. (XLSX 9 kb)

Additional file 5: Text 1 Amino acid sequences of grape, maize, rice and *Arabidopsis* HD-Zip proteins used for phylogenetic analysis. (TXT 82 kb)

Additional file 6: **Figure S2.** Conserved motifs in grape HD-Zip proteins. (TIFF 16924 kb)

Additional file 7: **Table S4.** Synteny regions between grape HD-zip genes. (XLSX 9 kb)

Additional file 8: **Table S5.** Synteny regions HD-zip genes between grape and *Arabidopsis. (XLSX 11 kb)*

Additional file 9: **Figure S3.** Expression of HD-Zip genes in ovules of PN and TS. The qRT-PCR data were analyzed by three independent replicates, and each duplication was repeated in triplicate, standard deviations are shown with error bars. Significant (P < 0.05) differences are indicated by an asterisk. The graphs are arranged as classification in phylogenetic tree. (TIFF 4356 kb)

Additional file 10: **Figure S4.** Expression of HD-Zip genes in somatic embryo of TS. PEM: proembryogenic masses; GE: globular embryo; HE: heart embryo; TE: torpedo embryo; CE: cotyledon embryo. All qRT-PCR experiments were employed with three biological duplications, and each duplication was repeated in triplicate, standard deviations are shown with error bars. Significant (P < 0.05) differences were used. The graphs are arranged as classification in phylogenetic tree. (TIFF 4628 kb)

Additional file 11: **Figure S5.** Expression of HD-Zip genes in different tissues in TS. All qRT-PCR experiments were employed with three biological duplications, and each duplication was repeated in triplicate. Significant (P < 0.05) differences were used. The graphs are arranged as classification in phylogenetic tree. (TIFF 3199 kb)

Additional file 12: **Figure S6.** ORF sequence of VvHDZ28 and its encoding protein. Protein under black line represent the HD region, and red line represent the LZ region. (TIFF 49 kb)

## Abbreviations

AbA: Aureobasidin A
ABRE: abscisic acid responsive element
CE: cotyledon embryos
DAF: day after flowering
GARE: gibberellin-responsive element
GE: globular embryo
HE: heart embryos
LTR: low temperature responsive element
MBS: MYB-binding site
PEM: Proembryogenic masses
PN: Pinot noir
SD: synthetically defined medium
TCA: salicylic acid responsiveness element
TE: torpedo embryos
Trp: tryptophan
TS: Thompson Seedless
X-ɑ-gal: 5-bromo-4-chloro-3-indolyl-a-D-galactopyranoside
Y2H: yeast two hybrid system strains
