# Supplementary figures and images for: Evolution and expression analysis reveal the potential role of the HD-Zip gene family in regulation of embryo abortion in grapes (Vitis vinifera L.)
Source: BMC Genomics. 2017 Sep 21;18:744. doi: 10.1186/s12864-017-4110-y (PMC5609062; doi:10.1186/s12864-017-4110-y)

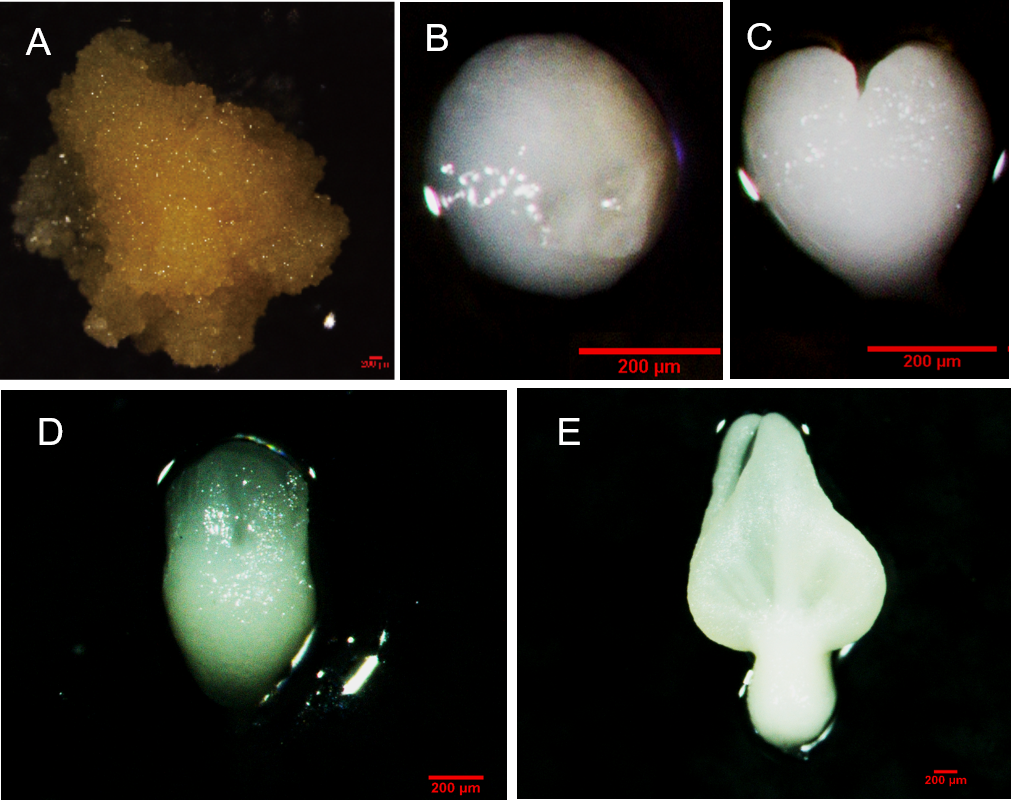

Supplement: Supplementary file 2 — Figure S1. Somatic embryo of Thompson Seedless at different stages. A: proembryogenic masses, PEM; B: globular embryo, GE; C: heart embryo, HE; D: torpedo embryo, TE; E: cotyledon embryo. (TIFF 2316 kb) [file 12864_2017_4110_MOESM2_ESM.tif]

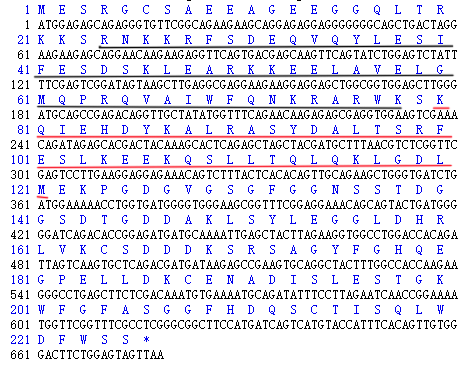

Supplement: Supplementary file 12 — Figure S6. ORF sequence of VvHDZ28 and its encoding protein. Protein under black line represent the HD region, and red line represent the LZ region. (TIFF 49 kb) [file 12864_2017_4110_MOESM12_ESM.tif]
